# Authors’ Response to Peer Reviews of “Waiting Time and Patient Satisfaction in a Subspecialty Eye Hospital Using a Mobile Data Collection Kit: Pre-Post Quality Improvement Intervention”

**DOI:** 10.2196/40453

**Published:** 2022-08-09

**Authors:** Mathew Mbwogge, Nicholas Astbury, Henry Ebong Nkumbe, Catey Bunce, Covadonga Bascaran

**Affiliations:** 1 International Center for Eye Health London School of Hygiene & Tropical Medicine London United Kingdom; 2 Africa Eye Foundation Cameroon Yaoundé Cameroon; 3 Research Data & Statistics Unit The Royal Marsden NHS Foundation Trust London United Kingdom

**Keywords:** Waiting time, waiting list, patient satisfaction, quality improvement, clinical audit, ophthalmology, patient-centered care

*This is the authors’ response to peer-review reports for “Waiting Time and Patient Satisfaction in a Subspecialty Eye Hospital Using a Mobile Data Collection Kit: Pre-Post Quality Improvement Intervention.”*

## Round 1 Review

Dear Reviewer,

We start by thanking the editorial team for their expression of interest in the topic and the reviewers in helping to move the paper forward. We wound also like to thank you and the reviewers for the input in improving our work. The reviewer comments were quite constructive, and we ensured that the best possible clarifications and responses are provided. Kindly find our responses embedded in the point-based comments. We hope that our responses/clarifications will be satisfactory and that the reviewed manuscript will be of acceptable standard.

### Reviewer T [1]

#### General Comments

This article [2] studies the waiting time and patient satisfaction in a subspecialty eye hospital in Cameroon. It is a matter of fact that hospital-waiting time is a major concern in many other countries, but it is important that this paper concentrates on Cameroon. Moreover, the article mentions the use of a mobile data collection kit conducting pre-post quality improvement intervention. The article can be characterized quite innovative and offers a significant connection of theory with practice.

It would be quite interesting if the authors mention the reasons why they chose Cameroon and refer to some similar recent research. Although, it is clear why this project is necessary to be studied. The objectives of the study are clear and combine waiting time and satisfaction, 2 important factors for the increase of quality-of-life indicators. The methods that were used are suitable and adequate for this project and the authors follow a correct pathway for the implementation of their work.

The research leads to the result that the use of plan-do-study-act (PDSA) led to a borderline significant reduction of 65.4 minutes in waiting time over 6 weeks and an insignificant improvement in satisfaction, suggesting that quality improvement efforts have to be maintained over a considerable period to be able to produce significant changes. The study provides a good basis for quality improvement in limited-resource settings making use of block appointment systems, with comprehensive subspecialty eye care services. We recommend shortening the patient pathway and other measures including a phasic appointment system, automated patient time monitor, robust ticketing, patient pathway supervision, standard triaging, task shifting, doctor consultation planning, patient education, and additional registration staff.

#### Specific Comments

##### Minor Comments

1. It would be quite interesting if the authors mention the reasons why they chose Cameroon and refer to some similar recent research.

RESPONSE: This is the first study targeting the improvement of waiting time and satisfaction in ophthalmology in Cameroon. Other quality improvement studies were found [3-5], including those that aimed at investigating patients’ satisfaction with the quality of health care services [6] and the undertaking of antiretroviral treatment [7]. Our choice of setting has been explained and similar studies are alluded to. Kindly refer to the first paragraph of the study rationale (highlighted in yellow).

2. The description of the problem can be enriched with some more information.

RESPONSE: We have now phrased the problem as suggested.

3. A justification of why this research method was chosen can be an extra asset for this interesting work.

RESPONSE: Thanks for this suggestion. The before and after study designs making use of the model for improvement have been widely reported [8]. We prioritized the simple pre-post study design because we wanted to make the study as close to reality as possible; second, it was a single-center study, which ruled out the possibility of making use of a control group. We have also shown that the PDSA is a widely used model in quality improvement studies [9-11]. This has been explained (highlighted in yellow) in the *Approach to Impact Assessment* subsection per the SQUIRE reporting guidelines for quality improvement studies [12,13].

4. I also believe that the authors have used too many references than normal in a paper. They might decrease the number of references and stay in the most appropriate range. Too many citations are used in this paper. Most journals recommend no more than 40 references.

RESPONSE: We thank you for raising this point. Although more explanations and justifications from reviewers simply meant more references to substantiate our clarifications, we have reduced the total number of citations. We believe we adhere to the journal guidelines and are not aware of any restrictions on the number of citations in JMIR journals. Should this be the case, we will be happy to review. Moreover, this suggestion seems to conflict with the editorial suggestion for more citations.

### Reviewer BK [14]

#### General Comments

In this paper, the authors aimed at improving patient waiting times and satisfaction through the use of PDSA quality improvement cycles. It is an interesting practical study. However, there are some major issues that need to be addressed by the authors. The following comments can help the authors improve the manuscript.

#### Specific Comments

##### Major Comments

1. In the Abstract and Methods sections, what does “ODK” stand for?

RESPONSE: Open Data Kit (ODK) is a mobile data collection app [15]. This has now been clarified (highlighted in green).

2. I suggest moving the problem description to the study rational as the first paragraph of this section.

RESPONSE: While we thank the reviewer for their comment, we wish to remind them that we followed specific reporting guidelines [12,13] when reporting the results. We decided to maintain this text as it is.

3. In the methods section, the contents related to the data collection need to be expanded to include the type of data that were collected by data collectors.

RESPONSE: We provided details of data collection under the subsection titled “Attributing Results to the Intervention” in the methods section (highlighted in green) based on the reporting guidelines mentioned earlier [12,13].

4. The methods of data collection should be explained clearly.

RESPONSE: We thank the reviewer for emphasizing on this. We wish to remind that we strictly followed the SQUIRE reporting guidelines because evidence suggests that quality improvement interventions are often poorly reported [16]. Two data collectors were purposely recruited for the study (see the “Data Collectors” subsection). They randomly approached participants at the point of entry and enrolled those who consented. Kindly refer to the “Attributing Results to the Intervention” subsection for more details (highlighted in green).

5. In the Results section (page 11), the authors said, “The first 7 changes were implemented, which includes…” and “Five of the originally proposed changes could not be implemented due to…” I think it is better if the authors either change the wording of the sentences or provide a complete explanation of the all changes. Then, the authors can explain which strategy was implemented and which one was not implemented.

RESPONSE: We remain grateful for this comment. Our explanation of implemented changes on page 12 under “Intervention Time Line,” which have now been modified, follows a complete list of all proposed changes found on page 6 under the “Changes Proposed” subsection, modified as well (text highlighted in green) as suggested.

6. In the Results section (*Unintended Outcomes* subsection), the authors noted the following: “…the intervention appeared to have affected women adversely…” This section needs further explanations about the possible reasons for such an unintended outcome.

RESPONSE: We appreciate the reviewer’s suggestion for more details. We further investigated why that was and found that the increase in waiting time was specific to the 15-24–year age group among women who were recruited after the intervention. We have now provided an explanation to that effect (highlighted in green) in the *Unintended Outcomes* subsection.

7. I am wondering whether all changes were implemented at the same time or they were implemented one by one. In case of the second approach, the impact of each strategy on changing waiting times and improving patient satisfaction could be investigated separately and compared with other strategies.

RESPONSE: Given the time constraint, we implemented all the changes together. We have added a phrase under the “Intervention Time Line” subsection on page 12, to make this clear (highlighted in green).

8. What were the possible reasons for non-significant increase in patient satisfaction while the waiting time was improved?

RESPONSE: We provided an explanation for this in the last paragraph of the “Association between patient satisfaction and waiting time” subsection of the Discussion. Kindly see text highlighted in green on page 21.

9. As the authors noted in the *Strengths and Limitations* section, the sample size was relatively small. However, they need to explain more why they did not reach a larger sample size. What were the main limitations?

RESPONSE: We worked with a limited sample size given the limited timeframe and data collectors. By its very nature, the time motion study required that data collectors record the time spent at each service point (kindly see flow chart on page 11) while shadowing, from entry through exit. This led to a maximum enrollment of 2 participants per day per data collector, provided they still had arrivals after finishing with the first participant. This has now been clarified in the text highlighted in green in the *Strengths and Limitations* subsection.

##### Minor Comments

1. Multimedia appendices were not available to me.

RESPONSE: Dear Reviewer, we know not why that was but our online manuscript management profile indicates that there were 2 supplementary files attached as Multimedia Appendices.

2. Any survey instruments or questionnaires used for measuring patient satisfaction need to be added to the manuscript.

RESPONSE: The data collection form downloaded from the ODK data form validation app [17] has now been uploaded as a Multimedia Appendix.

### Anonymous [18]

I have completed the statistical review of this manuscript, which is well-organized and presented. However, the following suggestions will help improve the quality of this manuscript.

1. Is it a proof-of-concept–type study? Kindly add the time period of this study.

RESPONSE: This was not a proof-of-concept study. We have now included a statement to clarify the period in the first sentence of the “Study Setting” subsection in the Methods section (highlighted in pale blue).

2. Kindly do not use word “subjects” for study participants. You can simply use either “participants” or “patients.”

RESPONSE: This has been amended accordingly.

3. No power calculation rationale was provided in this report, so these results cannot be generalized.

RESPONSE: We thank the reviewer for this comment. We have amended the text (highlighted in pale blue) in the first paragraph of the study conclusion as well as in the Abstract’s conclusion statement to reflect this.

4. Authors must include statements regarding the statistical software to perform data analysis and what level of statistical significance was used for hypothesis testing.

RESPONSE: We thank the reviewer for pointing this out. We have now included some text and made amendments (highlighted in pale blue) in the Abstract’s methods statement and the “Data Analysis” subsection on page 8, to this effect.

5. Authors must add more clarity to the “Logistic regression with reported…” statement as odds ratios with 95% confidence intervals are calculated from the logistic regression. What is the point of margins plot in this case? What other covariate were adjusted in the logistic regression? Kindly provide proper details with more clarity.

RESPONSE: We have added some text (highlighted in pale blue) under the “Association of Waiting Time and Satisfaction” subsection under “Data Analysis,” as suggested.

6. Table 1, the cohabiting group can be merged with the married group. Add “years” in brackets next to “Age.” Arrival time can also be sensibly presented with fewer meaningful categories.

RESPONSE: Thanks for the suggestion. The table has been amended accordingly.

## Round 2 Review

Dear Reviewer,

We thank you for your additional comments and commitment to improving the paper. While we note that the editorial comments were already addressed in the previous round, as seen below, kindly refer to the “external peer-review report” section for our responses to additional reviewer comments.

### External Peer-Review Reports

#### Reviewer BK [14]

##### General Comments

I appreciate the authors for their time and efforts to implement our suggestions. However, some issues need further attention.

1. The Introduction section started with the problem description. This section usually comes later and after describing the background. Hence, the coherence of the paragraphs should be revised. Moreover, the current subheadings in the Introduction section seem unnecessary and the authors can remove or reduce them.

RESPONSE: This has been amended accordingly.

2. As the authors said, they implemented all the changes together. However, each strategy or change might have a different impact on changing waiting times and improving patient satisfaction, which was worth investigating. If the authors did not do so, it is better to add this point to the *Strengths and Limitations* section.

RESPONSE: We have added this in the *Strengths and Limitations* section to highlight this point.

## Round 3 Review

### Reviewer BK [14]

Dear Reviewer,

We thank you for your comments. We note with regret that our responses to your comments were already submitted on April 11, 2022, but for some reasons unknown, we got another email notifying us of the same comments. Kindly see below the responses to your comments that were already submitted.

#### General Comments

I appreciate the authors for their time and efforts to implement our suggestions. However, some issues need further attention.

1. The Introduction section started with the problem description. This section usually comes later and after describing the background. Hence, the coherence of the paragraphs should be revised. Moreover, the current subheadings in the Introduction section seem unnecessary and the authors can remove or reduce them.

RESPONSE: This has been amended accordingly.

2. As the authors said, they implemented all the changes together. However, each strategy or change might have a different impact on changing waiting times and improving patient satisfaction, which was worth investigating. If the authors did not do so, it is better to add this point to the *Strengths and Limitations* section.

RESPONSE: We have added this in the *Strengths and Limitations* section to highlight this point.
